# Genome-wide analysis to uncover how *Pocillopora acuta* survives the challenging intertidal environment

**DOI:** 10.1038/s41598-024-59268-0

**Published:** 2024-04-12

**Authors:** Rosa Celia Poquita-Du, Danwei Huang, Peter A. Todd

**Affiliations:** 1 Experimental Marine Ecology Laboratory, S3 Level 2, Department of Biological Sciences, National University of Singapore, 16 Science Drive 4, Singapore, 117558 Singapore; 2 Lee Kong Chian Natural History Museum and Tropical Marine Science Institute, National University of Singapore, 2 Conservatory Drive, Singapore, 117377 Singapore

**Keywords:** Local adaptation, Genome-wide association study, Intertidal, Extreme environment, Ecology, Evolution

## Abstract

Characterisation of genomic variation among corals can help uncover variants underlying trait differences and contribute towards genotype prioritisation in coastal restoration projects. For example, there is growing interest in identifying resilient genotypes for transplantation, and to better understand the genetic processes that allow some individuals to survive in specific conditions better than others. The coral species *Pocillopora acuta* is known to survive in a wide range of habitats, from reefs artificial coastal defences, suggesting its potential use as a starter species for ecological engineering efforts involving coral transplantation onto intertidal seawalls. However, the intertidal section of coastal armour is a challenging environment for corals, with conditions during periods of emersion being particularly stressful. Here, we scanned the entire genome of *P. acuta* corals to identify the regions harbouring single nucleotide polymorphisms (SNPs) and copy number variations (CNVs) that separate intertidal colonies (*n* = 18) from those found in subtidal areas (*n* = 21). Findings revealed 74,391 high quality SNPs distributed across 386 regions of the *P. acuta* genome. While the majority of the detected SNPs were in non-coding regions, 12% were identified in exons (i.e. coding regions). Functional SNPs that were significantly associated with intertidal colonies were found in overrepresented genomic regions linked to cellular homeostasis, metabolism, and signalling processes, which may represent local environmental adaptation in the intertidal. Interestingly, regions that exhibited CNVs were also associated with metabolic and signalling processes, suggesting *P. acuta* corals living in the intertidal have a high capacity to perform biological functions critical for survival in extreme environments.

## Introduction

Many coral reef ecosystems worldwide are threatened by both impacts of climate change and unprecedented rates of urbanization^1,2^. Coastal development has resulted in extensive transformation of natural shorelines through the installation of artificial structures, such as seawalls and other defences, to prevent coastal inundation^3^. As the intertidal surfaces of seawalls are typically species-poor, there is a growing interest in enhancing the ecological value of these structures via transplantation of reef-building corals^4–6^. However, the seawall environment in the intertidal zone is generally suboptimal for hard coral establishment^7–9^. In particular, the conditions during periods of emersion, with associated exposure to desiccation risk and high light levels and temperatures, can be especially detrimental for corals^10,11^.

Spatial structuring of hard coral communities is generally attributed to the surrounding environment in which presence or absence of species reflects their tolerance limits^12,13^. This is commonly exhibited as depth ranges, but also applies to salinity, wave energy, and sediment tolerances^9,14–16^. It is assumed that organisms living in extreme environments are more stress-resistant^17,18^. While previous reports have documented rich coral communities thriving in intertidal nearshore habitats^8,9,14^, few studies have attempted to determine how these intertidal corals survive such stressful conditions. In particular, Zhang et al.^19^ have shown that selective sweeps occur among intertidal coral populations in which gene ontology categories associated with regulation of apoptosis were significantly enriched.

Generally, intertidal species exhibit various massive morphological forms (e.g. massive, encrusting, foliose) but branching corals, including acroporids and pocilloporids, also exist in these areas^9,15,20^. In particular, the pocilloporid species *Pocillopora acuta* can inhabit a relatively wide range of habitats, including the upper subtidal and lower intertidal zones^20–23^. In Singapore, this species has been observed growing well in atypical coral habitats such as the base of seawalls and the sides of marina pontoons^24^. However, they exhibit among-genotype variation in physiological and molecular responses to changing conditions^4,25–27^. For instance, a previous experiment showed this species can withstand 2 h of emersion, but only certain genotypes were able to survive longer emersion periods^4^. This indicates substantial intraspecific variability in stress tolerance in *P. acuta* that is likely driven by genetic variation among genotypes.

To date, most studies on genome-wide DNA polymorphisms in corals aimed to determine population structure and genetic connectivity^28–31^ while the structural and functional effects of these variants are rarely examined. Nevertheless, some research has highlighted genomic signatures of adaptation in corals to challenging conditions [e.g. ^32–34^. In particular, Bay and Palumbi^33^ showed temperature adaptation in *Acropora hyacinthus* colonies living in a backreef pool that experiences highly-variable temperature. They found signals of selection on coding regions that interact with heat-shock proteins, as well as those that involved in antioxidant defense and apoptosis. Another study on *Acropora* corals by Cooke et al.^32^ showed selective sweeps for colonies found in inshore habitats of the Great Barrier Reef. The sweep loci are associated with osmotic regulation and skeletal development, which were linked to challenging water quality conditions (e.g. hyposaline) surrounding inshore coral populations.

Expression levels of genes are known to be influenced by many factors including the structural location and nature of DNA polymorphisms within the genome, which in turn can affect the associated functions that control various biological processes^35^. Generally, such inferences are obtained by identifying single nucleotide polymorphisms (SNPs), however, more recently, copy number variation (CNV) of putative genes has also emerged as an important genetic mechanism that can ultimately influence an organism's adaptive capacity to environmental change^36^. For example, some hypoxia-associated genes were identified to exhibit large copy variation among colonies of the massive *Porites lutea*, which is known to tolerate highstress environments such as the intertidal zone^37^. Although contributions of CNVs and SNPs to phenotypic variation are largely independent, they may act in concert with each other^38,39^. While knowledge about the effect of both types of variation co-existing in the same region is limited, it has been shown that co-existent CNVs can magnify gene-trait association power of SNPs^40^.

Studies aiming to identify genomic variations and their corresponding biological functions are fundamental to uncovering variants underlying trait differences among and within-coral species. Findings can also uncover genes that are potentially under selection which can suggest adaptation to certain environments^41^. Here, we hypothesize that (1) there is significant genomic variation, in the form of single nucleotide polymorphisms (SNPs), that differentiates intertidal and subtidal *P. acuta* colonies and, (2) identified regions harbouring SNPs also exhibit CNVs. Lastly, we aim to characterize the functional relevance of these genomic variations (i.e. SNPs and CNVs) in the context of the intertidal/subtidal environments they were sampled from.

## Materials and methods

### Sample collection

**Figure 1:**
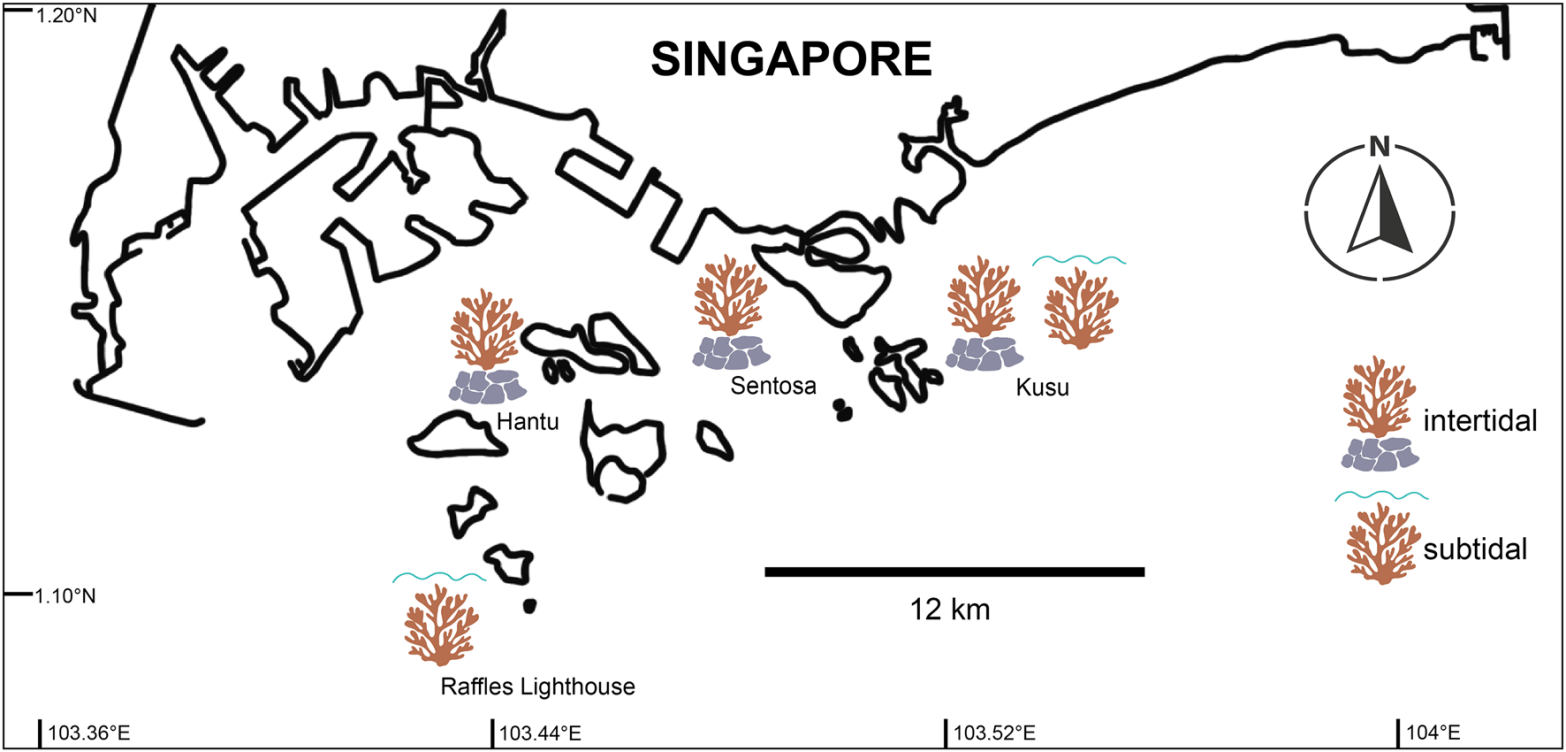
Map showing all sites within Southern Islands of Singapore where *P. acuta* colonies were collected from intertidal (Pulau Hantu, Kusu Island, Sentosa Island) and subtidal (Kusu, Raffles Lighthouse) areas.

Coral nubbins (~ 2 cm size) from different colonies of *Pocillopora acuta* found on the intertidal seawalls at Pulau Hantu (n = 8), Kusu (n = 5) and Sentosa (n = 5) (Fig. 1) were collected in duplicate during low tide periods. Colonies of *P. acuta* were identified following Poquita-Du et al.^42^. Specifically, these corals were collected at the base of the seawalls and are submerged during high tide up to a depth of 2 to 3 m, depending on the location^43^. For *P. acuta* colonies in subtidal areas (~ 6 m depth), duplicate fragments were collected from different colonies at Kusu (n = 12) and another site: Raffles Lighthouse (n = 9) underwater using SCUBA as they are fully-submerged even during low tide. Collection sites and number of samples depended on the presence of *P. acuta* and colonies having approximately the same maximum diameter size (~ 20 cm).

All 78 coral nubbins (samples hereafter) of *P. acuta* (39 colonies × 2 replicates) were immediately fixed in RNAlater and kept in an icebox while being transported to the laboratory. All samples were kept in a − 80 °C freezer until DNA extraction (conducted within a week of collection).

### DNA isolation, whole genome sequencing and data quality check

Genomic DNA (gDNA) was extracted using Qiagen DNeasy Blood and Tissue DNA extraction kit following the manufacturer’s protocol with an extra step of RNA removal using RNase solution (Promega). The procedures for library preparation and sequencing followed Azenta Life Sciences (China). Briefly, the library preparation was carried out using ND607 VAHTS Universal Library Kit V3 following the manufacturer’s protocol. For each sample, 100 ng genomic DNA was randomly fragmented to < 500 bp by sonication (Covaris S220). The fragments were treated with End Prep Enzyme Mix for end repairing, 5’ phosphorylation and dA-tailing in one reaction, followed by a T-A ligation to add adaptor to both ends. Size selection of adaptor-ligated DNA was then performed using beads and fragments of ~ 470 bp (with approximate insert size of 350 bp) were recovered. Each sample was then amplified by polymerase chain reaction (PCR) using P5 and P7 primers, with both primers carrying sequences which can anneal with flow cell to perform bridge PCR and, P5/P7 primer carrying index allowing for multiplexing. The PCR products were subsequently purified using magnetic beads, validated using an Qsep 100 (Bioptic, Taiwan, China), and quantified by Qubit 3.0 Fluorometer (Invitrogen, Carlsbad, California, USA).

The libraries with different indices were multiplexed and paired-end (pe) sequencing was performed on the Illumina NovaSeq 6000 platform according to the manufacturer’s instructions (Illumina, San Diego, California, USA) to generate 150 bp pe reads (30 × coverage). Image analysis and base calling were conducted using the NovaSeq Control Software (NCS) + OLB _ GAPipeline-1.6 on the NovaSeq instrument. Adapter sequences, PCR primers, content of N bases more than 10% and bases of quality lower than 20 were removed with cutadapt (V1.9.1)^44^.

### Sequence alignment and SNP detection

An annotated draft genome of *P. acuta*^45^ was used as the reference genome assembly in this study. While the *P. acuta* genome assembled by Vidal-Dupiol et al.^45^ was based on Illumina short-read sequencing, the quality is acceptable (BUSCO completeness of 89.4% and 91.4% based on Metazoa and Eukaryota datasets, respectively) and it was the only available *P. acuta* genome during the analysis. In support, the colony used to generate the *P. acuta* genome by Vidal-Dupiol et al.^45^was collected from the same region (i.e. Indonesia) not far from where our samples were collected (Singapore), therefore, it is an ideal reference genome for our study.

Sequence alignment was performed by individually mapping pe reads for all samples against the reference assembly using Burrowers-Wheeler Aligner (BWA, v 0.7.13) maximal exact match (mem) algorithm^46,47^. The reference assembly was indexed prior to mapping with sample sequences using the function ‘*bwa index’*. Unmapped and duplicate mapped reads were subsequently removed using SAMtools (v 1.10)^48^. Initial identification of genotype likelihoods at each genomic position, variant calling and subsequent normalization of variants were performed using bcftools v1.11^49^. The outputs were filtered to retain SNPs using Plink^50^. Further, SNPs that showed extended linkage disequilibrium (LD) in which a correlated (r^2^ = 0.5) pair of SNPs within a window of 50 kb were removed from the data set. In addition, markers that showed extensive deviation from Hardy–Weinberg equilibrium (HWE; *p* < 0.001), a missing rate of 1%, and low minor allele frequency (MAF) of 5% were excluded.

### Examining population structure and admixture proportions

To examine the genetic divergence between *P. acuta* colonies from two tidal environments, the fixation index (F_ST_) was estimated in Plink using the final output of SNPs generated above. Principal component analysis (PCA) was also performed in Plink and plotted in R^51^ to visualize the genetic structure of *P. acuta* colonies from different sites and tidal environment. Additionally, we assessed the proportions of ancestry for up to five potential K values, with computations of cross validation (CV) errors, using Admixture v1.3.0^52^ and visualized using custom python scripts.

### Association analysis and annotation of SNPs

Identification of specific regions harbouring SNPs that were significantly associated with intertidal vs. subtidal *P. acuta* colonies was performed using Plink^53^. Allele frequencies of quality-filtered SNPs were compared between intertidal and subtidal colonies using Fisher’s exact test to generate *p* values. SNPs with association *p* values of ≤ 0.01 were considered significantly associated with intertidal *P. acuta* colonies^50,54,55^. Results were visualized with a Manhattan plot using ggplot2 and qqman packages in R^51,56,57^.

Analysis for the distribution of all SNPs across genomic regions and structural annotation were performed using snpEff^58^. The output of the annotation run included translated protein sequences which were filtered to regions where SNPs were significantly associated with intertidal colonies using the sequence processing tool, ‘mothur’^59^. The output list of protein sequences obtained from the previous step were then aligned against the most updated Swissprot/Uniprot database using NCBI Blast (‘blastp’ command). Gene ontology (GO) enrichment analysis was subsequently performed using BiNGO (V 3.0.5; https://www.psb.ugent.be/cbd/papers/BiNGO/Home.html) available in Cytoscape (V 3.9.1, http://www.cytoscape.org/)^60,61^ by performing Hypergeometric test with Benjamin and Hochberg False Discovery Rate (FDR) multiple testing correction (significance level = 0.05) on the set of genes from the *blastp* output.

### Orthology and copy number variation

Orthogroups were predicted to help determine whether genomic variations that differentiate intertidal and subtidal *P. acuta* colonies were also driven by CNVs. OrthoFinder v2.5.4, a tool that implements the phylogenetic orthology inference method^62,63^, was used to predict orthogroups. Sequences from protein-coding regions harbouring all variants, including SNPs, insertions, and deletions, for both tidal environments (i.e. intertidal and subtidal) were used for the orthology analysis (default settings). Orthogroups that exhibited CNVs, particularly those that were present in both environments, were identified and the corresponding protein sequences belonging to these orthogroups were retrieved for alignment process. Using the BLAST built-in tool (*blastp*) in CLC Genomics Workbench (V 10.0.1), protein sequences from the different orthogroups were aligned against the most recent version of the UniProt SwissProt protein database (accessed 14 April 2022). BLAST hits that showed the highest number of HSP (high-scoring segment pair) were used to search for corresponding gene IDs and putative functions on UniProtKB^64^.

## Results

### Sequencing and alignment

Illumina sequencing resulted in an average of 56.1 million pe raw reads per sample (Supplementary data). After removal of low quality and adapter reads, 55.5 million pe reads per sample were retained, with 38.76% GC content and Q phred scores of 97% Q20. For each sample, an average of ~ 96% of quality-filtered sequences mapped to the *P. acuta* draft genome^45^.

### SNP detection and annotation

**Figure 2:**
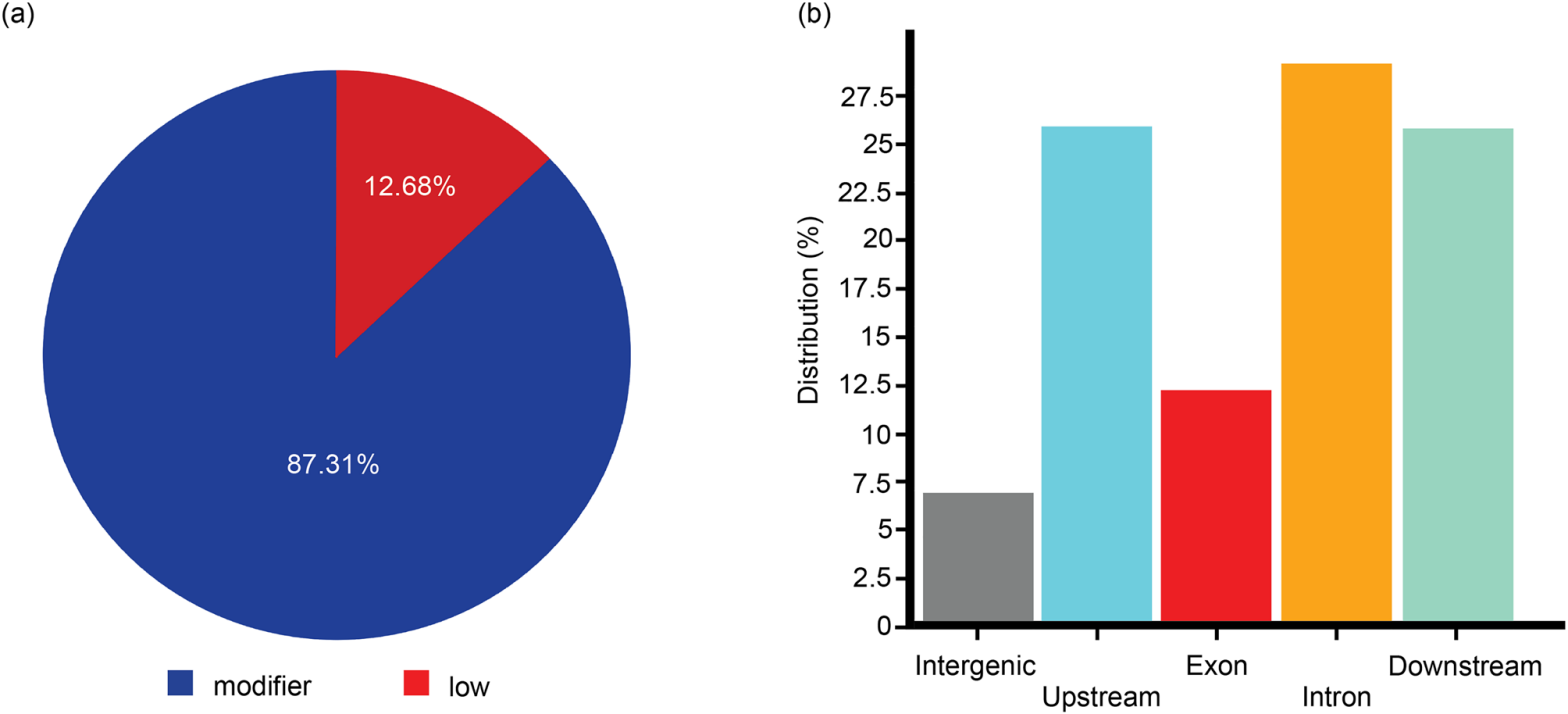
Structural annotation showing percentage of SNP effects by impact categories (**a**) and distribution of SNPs in different genomic regions (**b**).

Overall, there were 29,619,409 variants obtained from the variant detection analysis, with average genotyping rate of approximately 99%. These variants consisted of 25,773,468 SNPs, 1,345,237 insertions, and 2,500,704 deletions. Following a series of stringent filtering to obtain independent and high-quality SNPs, only 74,391 SNPs were retained. These SNPs were detected in varying locations within 386 genomic regions, of which 87.31% were categorized as modifiers (Fig. 2a), which usually are variants affecting non-coding genes. The SNPs were found in different regions within the *P. acuta* genome (Fig. 2b), including ~ 29% within introns and only ~ 12% in functional regions (i.e. exons).

### Population structure and admixture proportions

**Figure 3:**
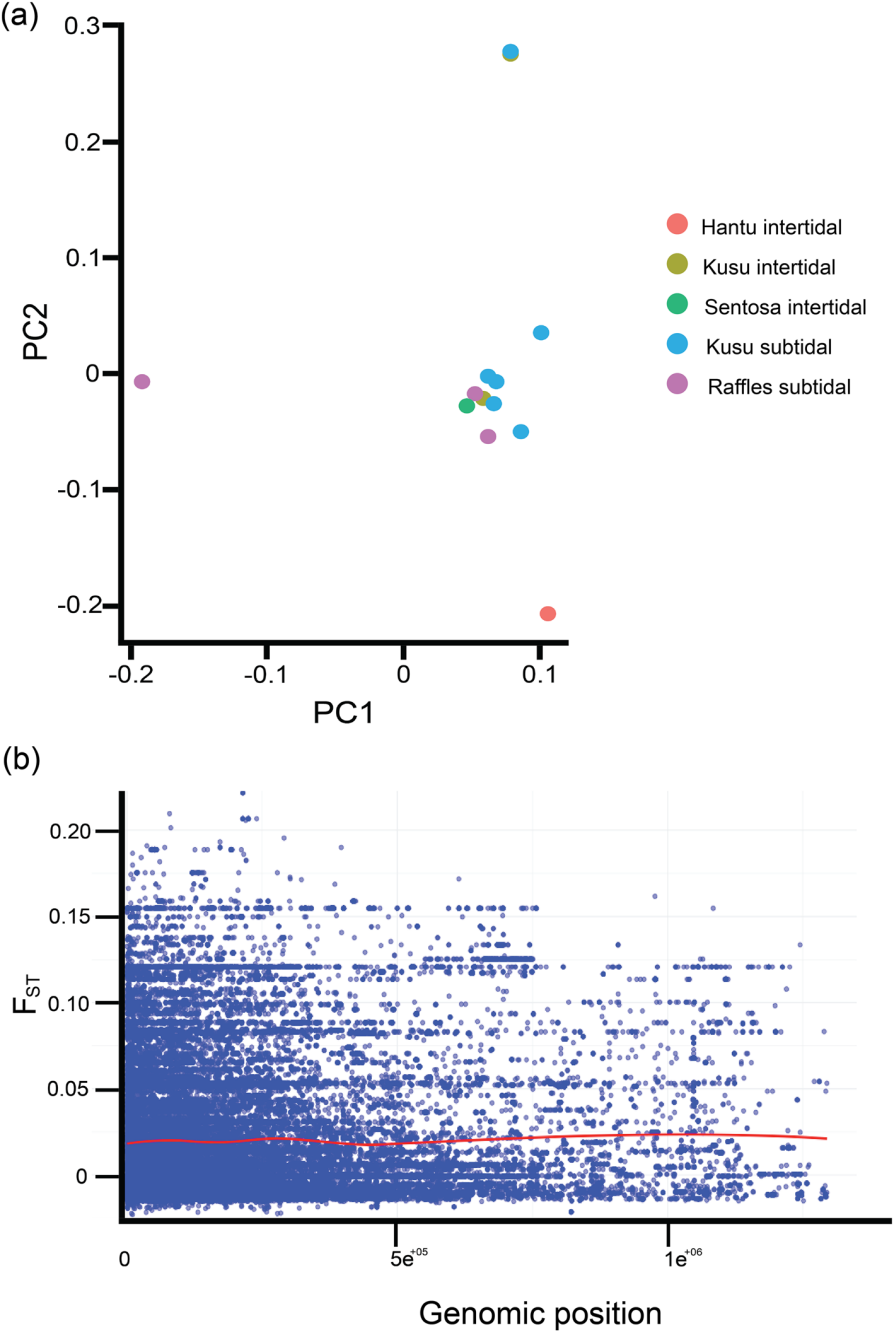
Genetic structure of *Pocillopora acuta* based on (**a**) Principal component analysis at the colony level and, (**b**) genetic divergence among colonies between the two tidal environments, showing estimates of F_ST_ for each identified SNP across various genomic positions.

**Figure 4:**
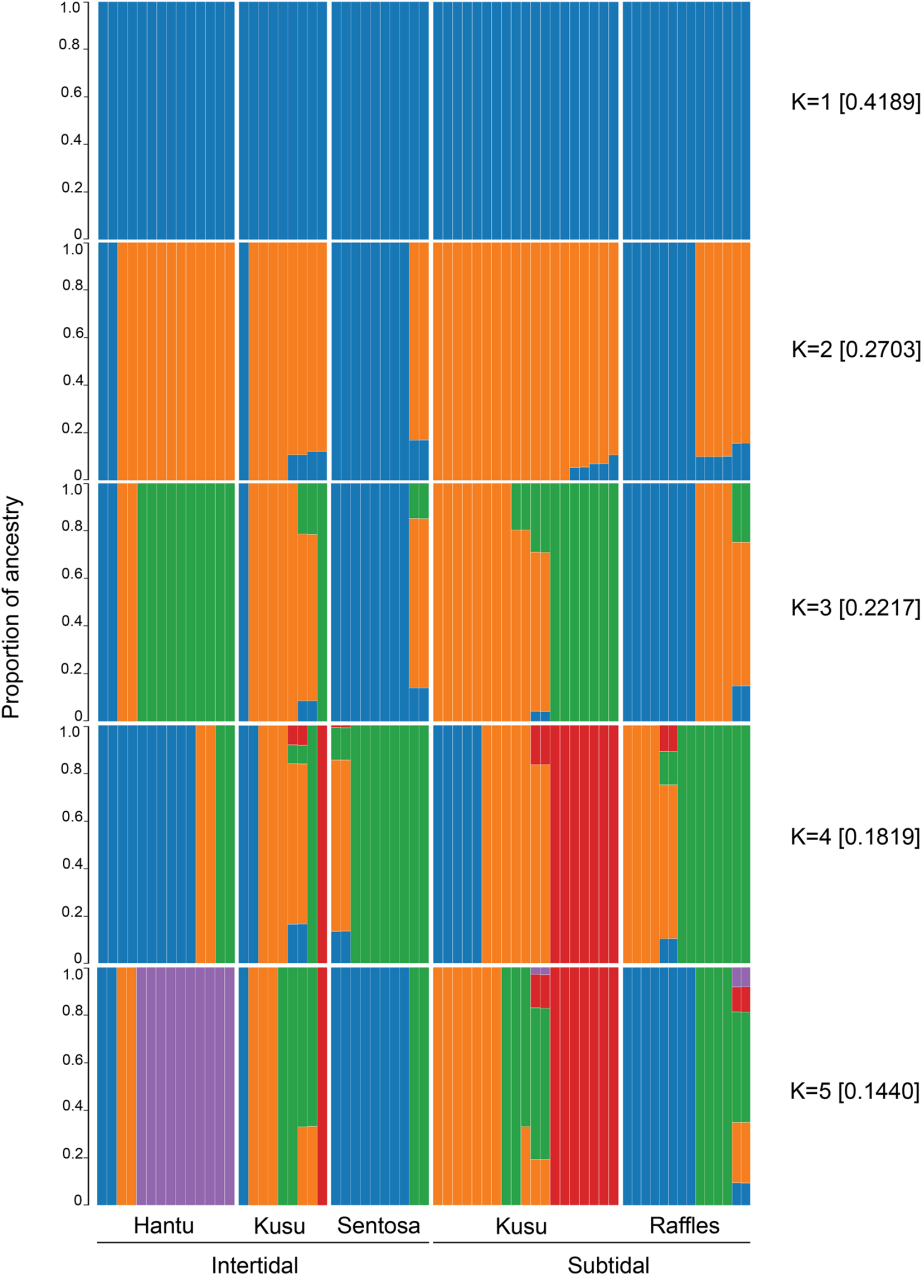
Proportion of ancestry calculated using Admixture, showing up to five potential K values and the corresponding CV errors in brackets.

Results from both PCA and Admixture (Figs. 3a and 4, respectively) showed no structuring based on site and tidal environments. F_ST_ values for all identified SNPs across various genomic positions (mean Global F_ST_ = 0.02) were generally low (Fig. 3b), indicating low genetic differentiation between *P. acuta* colonies found in the intertidal and subtidal environment.

### Association analysis

**Figure 5:**
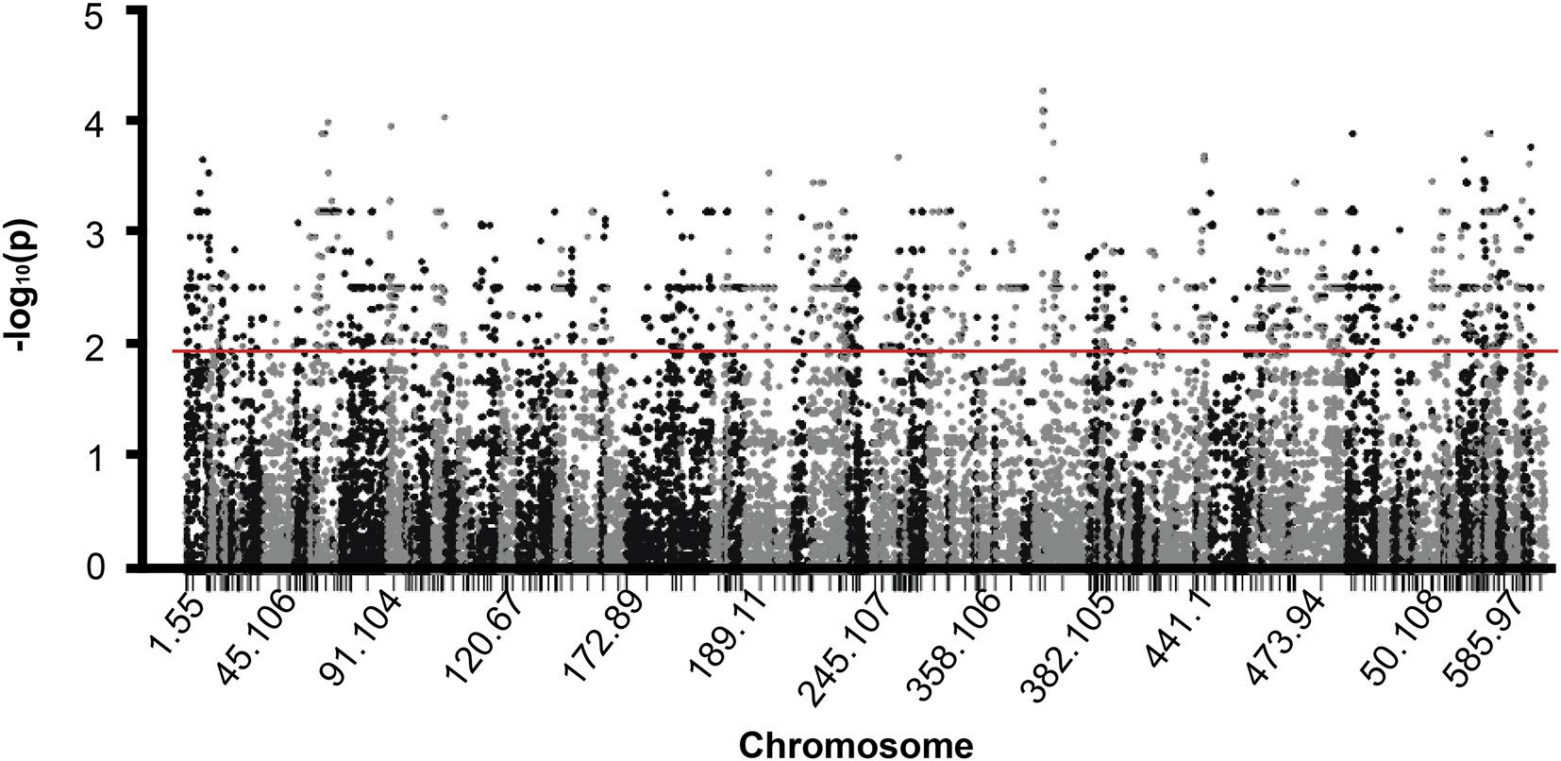
Manhattan plot showing allele frequencies of quality-filtered SNPs from different chromosomes. SNPs that were highly associated with intertidal colonies (*p* values of < 0.01) are indicated by the red horizontal line.

Out of 386 regions that harbour SNPs, 134 of them were found to be significantly associated with intertidal colonies (*p* values < 0.01; Fig. 5). A complete inventory of all the regions with SNPs and their specific locations are provided in the Supplementary Data.

### Gene ontology enrichment analysis for intertidal-associated SNPs

**Figure 6:**
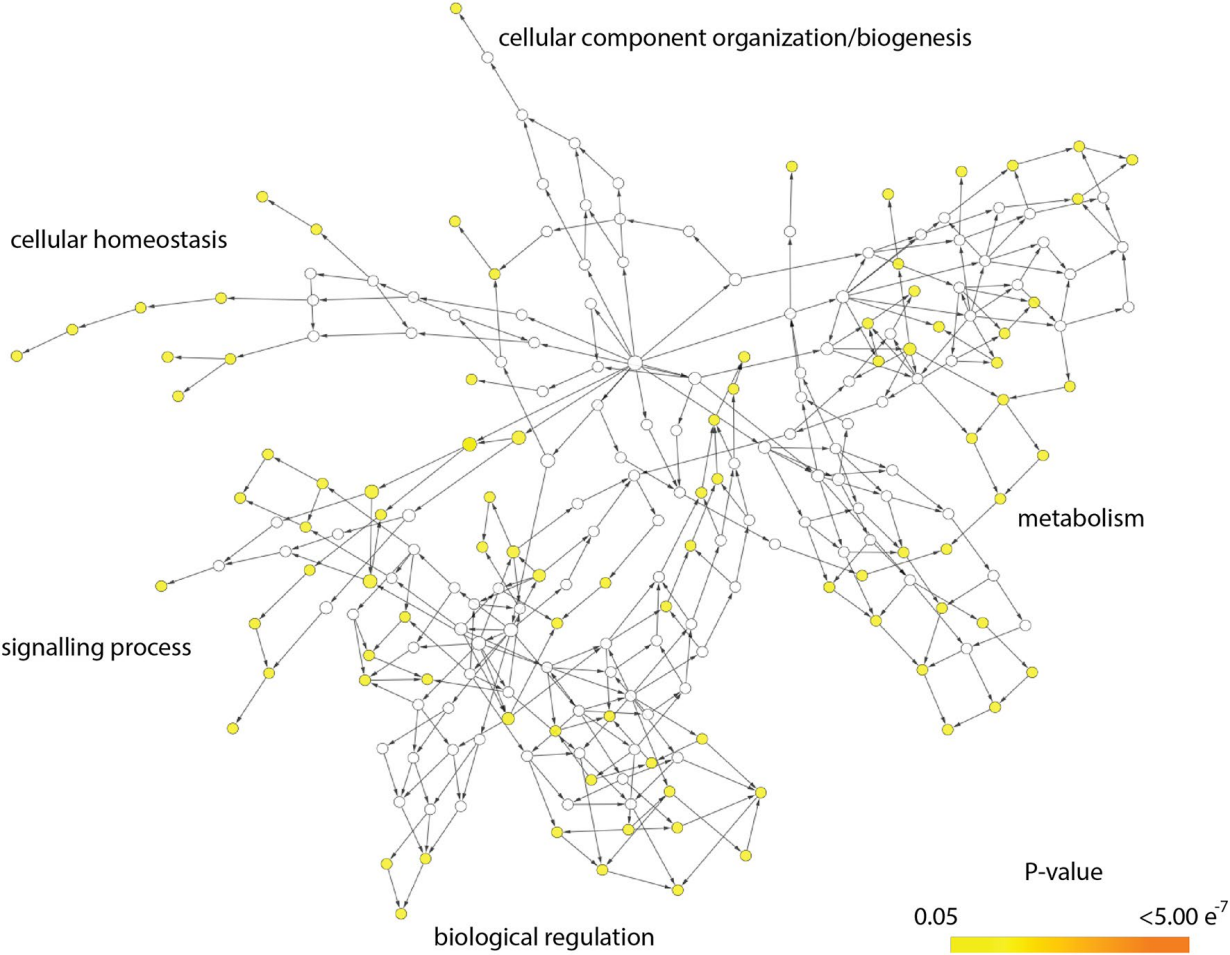
Gene ontology enrichment analysis of genes significantly associated with intertidal *P. acuta* colonies, showing a gene network with significantly overrepresented genes (shaded nodes; cut-off *p* value 0.05) under the biological processes category.

While the majority of detected SNPs highly associated with intertidal *P. acuta* colonies were observed in introns, some were found in exons and hence predicted to be functional regions. However, only a small portion (~ 28%) of these intertidal-associated SNPs could be annotated based on homologs from well-studied taxa which were used to search for GO terms under biological processes categories. Significant overrepresentation of GO was observed for genes that are associated with cellular homeostasis, metabolism, and signalling processes (Fig. 6; also refer to Supplementary data). Due to the interdependency of GO categories, whole branches for each GO hierarchy are shown in Fig. 6, but only coloured nodes that are farthest down the network were considered relevant (refer to^60^). In particular, the overrepresentation of categories—cellular homeostasis, metabolism, and signalling process—reflect the presence of genes associated with responses to glucose stimulus, fatty-acid beta-oxidation, and regulation of mitogen-activated protein kinase (MAPK) and nuclear factor-kappa B (NF-kappaB) cascades.

### Orthology and copy number variation

OrthoFinder assigned 93.4% of the total number of genes (11,911 out of 12,755) into 2665 orthogroups, 570 of which were single copy orthogroups. Only genes that showed copy number differences > 2 between the two tidal environments are summarized in Table 1, with corresponding protein matches and putative functional profiles. The complete set of orthogroups that exhibit CNVs can be found in the Supplementary data.

**Table 1:** Summary of genes that exhibit copy number variations across two tidal environments, intertidal and subtidal, ordered by descending variance.

Orthogroup	Gene ID	Protein	Intertidal	Subtidal	Variance	Functional profile
OG0000017	*TMTC4	Protein O-mannosyl-transferase	27	1	26	Protein O-linked mannosylation
OG0000033	*stan	Protocadherin-like wing polarity protein stan	22	1	21	Calcium-dependent cell–cell adhesion via plasma membrane cell adhesion molecules
OG0000042	*AKR1	Ankyrin repeat-containing protein	19	1	18	Palmitoyltransferase specific for casein kinase 1
OG0000200	*Atp2b3	Plasma membrane calcium-transporting ATPase 3	9	1	8	Calcium ion export across plasma membrane
OG0000176	*Tmem145*	Transmembrane protein 145	3	7	4	G-protein-coupled receptor signalling pathway
OG0000293	n/a	n/a	2	6	4	n/a
OG0000294	n/a	n/a	2	6	4	n/a
OG0000304	*KCNF1*	Potassium voltage-gated channel subfamily F member 1	2	6	4	Regulation of ion transmembrane transport
OG0000444	*MIMI_R240*	Collagen-like protein 4	1	5	4	May participate in the formation of layer of cross-linked glycosylated fibrils
OG0000148	n/a	n/a	4	7	3	n/a
OG0000149	*ARHGEF7*	Rho guanine nucleotide exchange factor 7	4	7	3	Positive regulation of apoptotic process
OG0000237	*yomI	SPBc2 prophage-derived uncharacterized transglycosylase	6	3	3	Peptidoglycan metabolic process
OG0000238	n/a		3	6	3	n/a
OG0000377	*Cyb5rl*	NADH-cytochrome b5 reductase-like	2	5	3	Desaturation and elongation of fatty acids; cholesterol biosynthesis
OG0000384	*yraN*	UPF0102 protein	2	5	3	Nucleic acid binding
OG0000577	*SNRNP200	U5 small nuclear ribonucleoprotein 200 kDa helicase	4	1	3	mRNA splicing
OG0000584	n/a	n/a	1	4	3	n/a
OG0000604	*nma111*	Pro-apoptotic serine protease	1	4	3	Apoptotic process
OG0000605	*DNAI3*	WD repeat-containing protein 63	1	4	3	Negative regulation of cell migration
OG0000606	*adrb1*	Beta-1 adrenergic receptor	1	4	3	Adenylate cyclase-activating adrenergic receptor signalling pathway

Asterisks denote genes that exhibit higher number of copies for intertidal.

## Discussion

The environmental conditions surrounding intertidal areas are generally suboptimal for coral survival^8,11^, but several species have been observed to establish at the lower intertidal section of seawalls in Singapore including the common branching coral, *Pocillopora acuta*^9,15^. Here, we identified genomic regions harbouring SNPs that distinguish intertidal from subtidal *P. acuta* colonies. We also identified the presence of CNVs in all regions harbouring variants. The functional relevance of these regions, particularly coding regions, was further examined to gain insights into the possible involvement of these SNPs and CNVs in biological processes. Our results revealed that the genomic regions differentiating intertidal and subtidal colonies are mainly associated with cellular homeostasis, metabolism, and signalling processes, suggesting these are likely signatures of adaptation to extreme environments.

To account for population stratification and genetic ancestry, we examined the genetic structure and proportions of ancestry among *P. acuta* colonies studied here and found that there is no structuring based on site and tidal environment. While the five population divisions inferred when K = 5 had the lowest CV error, they do not coincide with the site or environmental groupings, implying that the limited structuring observed was not associated with these a priori subdivisions. Our observations align with findings in Afiq-Rosli et al.^28^, which also showed no clear among-site separation for *P. acuta* corals collected from reef sites within the Southern Islands of Singapore, including the collection sites in the present study [i.e., Hantu, Kusu, Satumu (Raffles Lighthouse)].

We found enrichment of genomic regions with SNPs that are linked to cellular homeostasis for intertidal *P. acuta* colonies. In particular, overrepresentation of responses to glucose stimulus was detected, likely due to corals relying on their endosymbionts for most of their daily energy budget through translocation of photosynthetic products (i.e. glucose)^65^. While photosynthesis is critical for corals to survive, it can contribute to the production of reactive oxygen species (ROS) which are known to be toxic for coral hosts and therefore need to be regulated to achieve homeostasis in the coral holobiont for efficient cellular functioning^66,67^. This is especially challenging for intertidal corals as they are exposed to high solar radiation and elevated temperatures for longer periods of time, which can destabilize the photosynthetic electron-transport chain of the endosymbiotic dinoflagellates, resulting in increased production of ROS^67–69^. Relatedly, there was a remarkably high number of gene copies coding for Protein O-mannosyl-transferase in the intertidal corals (Table 1). While the role of this protein is not known for corals, the function of O-mannosylation has been described for *Saccharomyces cerevisae* where it plays a role in solubilization of misfolded proteins during stressful conditions^70^. When there is an accumulation of misfolded proteins, the unfolded protein response (UPR) is triggered, however, prolonged UPR activation can cause production of ROS^71^. It is widely known that high levels of ROS can cause DNA damage which is detrimental to many organisms including corals^67,72,73^. Thus, high numbers of copies of genes coding for Protein O-mannosyl-transferase, which are protective against cell damage, may be beneficial for intertidal corals which are frequently exposed to extreme conditions.

Further, significant overrepresentation of genes associated with signalling pathways, MAPK and NF-kappaB were also detected in regions with SNPs associated with intertidal colonies. These pathways have been linked previously to oxidative stress in corals^73,74^. For instance, a significant activation of c-Jun N-terminal kinase (JNK, one of the major subgroups of MAPK gene family) has been shown to repress oxidative stress in *Stylophora pistillata* corals exposed to ultraviolet radiation (UVR) and thermal stress^75^. Similarly, genes associated with signal-transduction also displayed a relatively high number of copies for intertidal corals, particularly genes linked to Palmitoyltransferase specific to casein kinase 1 (*AKR1*) as well as calcium-dependent cell–cell adhesion via plasma (*stan*) and calcium ion export across the plasma membrane (*Atp2b3*). This suggests a highly coordinated signalling response to enable intertidal corals to adapt to extreme environments.

As with many other animals, metabolic activities in corals are known to be dynamic, responding to diel variations in environmental conditions such as light availability. For example, during daytime the coral’s diffusive boundary layer (DBL) can experience hyperoxia due to high photosynthetic activity^76^. Further, intense solar radiation can cause photoinhibition to endosymbionts, reducing their capacity to provide photosynthetically-fixed carbon to the coral host^65^. Our results show significant enrichment of metabolic processes associated with fatty acid beta-oxidation for intertidal corals. Fatty acids are important components of lipids in a wide range of living organisms, including corals^77^. Storage lipids play a crucial role in a coral’s metabolism, functioning as reserves to generate energy for survival during periods of adverse conditions^78,79^. A coral’s lipid stores can comprise up to ~ 40% of its total biomass, representing a substantial quantity of energy reserves^80,81^. The high occurrence of SNPs in metabolism-associated regions observed here implies a relatively high utilization of lipids for intertidal *P. acuta* corals, corroborating recent findings on metabolic plasticity of this species to withstand extreme environment^82^.

Overall, there is clear genomic variation within the species *P. acuta* living in two distinct tidal zones, suggesting population differentiation associated with these distinct environments. The genome-wide SNPs and CNVs reported here provide important insights into the biological functions (cellular homeostasis, metabolism, and signalling) that contribute to the capacity of *P. acuta* to withstand extreme conditions in the intertidal zone, but additional research on the expression of quantitative trait loci which involve direct association tests is required to confirm this. For instance, assessing lipid levels in colonies inhabiting two tidal environments can provide insights into the role of lipid metabolism in local acclimatization to different conditions. It is important to mention that this study is limited by the absence of *P. acuta* corals in the subtidal or intertidal area for some sites, thus, there is an unbalanced representation of the two tidal environments. Nevertheless, the genomic resources generated in this study can help facilitate discovery of functional variants associated with traits (e.g. stress tolerance) which can contribute towards active conservation efforts such as coral transplantation and selective breeding.

## Data Availability

Raw sequence reads and metadata are deposited at NCBI under BioProject PRJNA812628. Supplementary data supporting this article can be found online at https://zenodo.org/doi/10.5281/zenodo.6318484.
