# Author Correction: Genome-wide analysis to uncover how *Pocillopora acuta* survives the challenging intertidal environment

**DOI:** 10.1038/s41598-024-60738-8

**Published:** 2024-05-06

**Authors:** Rosa Celia Poquita-Du, Danwei Huang, Peter A. Todd

**Affiliations:** 1 Experimental Marine Ecology Laboratory, S3 Level 2, Department of Biological Sciences, National University of Singapore, 16 Science Drive 4, Singapore, 117558 Singapore; 2 Lee Kong Chian Natural History Museum and Tropical Marine Science Institute, National University of Singapore, 2 Conservatory Drive, Singapore, 117377 Singapore

Correction to: *Scientific Reports* 10.1038/s41598-024-59268-0, published online 12 April 2024

The original version of this Article contained an error in Reference 37, which was incorrectly given as:

Alderdice, R. et al. Deoxygenation lowers the thermal threshold of coral bleaching. Sci. Rep. 12, 18273 (2022).

The correct reference is listed below:

Alderdice, R. et al. Disparate inventories of hypoxia gene sets across corals align with inferred environmental resilience. Front. Marine Sci. 9, 834332 (2022)

The original Article has been corrected.

